# Nutritional modulation of the gut–reproductive axis: multi-strain probiotic blend on oxidative and seminal parameters in healthy male dogs

**DOI:** 10.3389/fvets.2026.1820546

**Published:** 2026-04-24

**Authors:** Caterina Losacco, Edmondo Ceci, Gianluca Pugliese, Vincenzo Tufarelli, Giulio Guido Aiudi

**Affiliations:** 1 Department of Precision and Regenerative Medicine and Jonian Area, Section of Veterinary Science and Animal Production, University of Bari Aldo Moro, Bari, Italy; 2 Department of Veterinary Medicine, University of Bari Aldo Moro, Bari, Italy

**Keywords:** diet, dog, fertility, probiotics, semen quality

## Abstract

This study investigated whether dietary supplementation with a multi-strain probiotic mixture (Slab51®, Sivomixx®) modulates systemic and seminal redox status and improves semen quality in healthy breeding dogs. Fourteen dogs were randomly assigned to receive either a control diet or the same diet supplemented with 400 billion CFU of lyophilized bacteria for 70 days, covering a complete spermatogenic cycle. Serum biochemical parameters and systemic antioxidant defences were evaluated at baseline and day 70. Semen was collected prior to supplementation and after 35 and 70 days of treatment. Probiotic supplementation significantly increased sperm concentration (*p* < 0.001) and improved key kinematic parameters, including average path velocity (VAP, *p* < 0.05), straight-line velocity (VSL, *p* < 0.05), curvilinear velocity (VCL, *p* < 0.01), amplitude of lateral head displacement (ALH, *p* < 0.05), and linearity (LIN, *p* < 0.001), whereas beat-cross frequency (BCF), straightness (STR), and static sperm percentage remained unchanged. These improvements were accompanied by a marked reduction in seminal reactive oxygen species (ROS) and a significant increase in biological antioxidant potential (BAP) and total antioxidant capacity (TAC), indicating enhanced seminal antioxidant capacity. Systemically, probiotic-treated dogs exhibited reduced blood urea nitrogen, triglycerides, and total cholesterol, together with significantly increased superoxide dismutase, catalase, glutathione peroxidase activities, and TAC (*p* < 0.001). In conclusion, these findings demonstrate that multi-strain probiotic supplementation enhances systemic and seminal antioxidant defences and promotes functional improvements in semen quality in healthy dogs, highlighting the biological relevance and practical implications for breeders and clinicians. However, further studies are ongoing to deeply elucidate the effects of probiotics on semen parameters and the relationship between probiotic-induced modulation of the gut microbiota and semen characteristics, with the aim of clarifying the role of the gut–testis axis in canine fertility and reproductive health.

## 1 Introduction

The expanding adoption of artificial insemination practices in dog’s breeding programs has drawn attention to the assessment of semen quality as an affordable marker of male reproductive efficiency, compelling the management and optimization of semen standard parameters (1–3). Accordingly, several studies have analysed the underlying pathways of subfertility or infertility and the conceivable approaches that may raise male dog reproductive efficiency.

Reproductive impairment in male can arise from factors impacting both sperm quality and quantity (4, 5). Notably, the quality of semen is dependent on a number of elements, including the suitable environment in which it is dispersed, which significantly affects sperm functions, development and survival. For instance, biochemical variations in the prostatic component of seminal fluid may impair sperm nutrition, resulting in poor viability, motility and morphological changes (6–9). Basically, seminal plasma contains endogenous, enzymatic, renewable, and exogenous, non-enzymatic, and non-renewable antioxidants, that help to maintain basal concentrations of reactive oxygen species (ROS) and to counteract and protect spermatozoa from ROS-mediated injury (3, 10–13). Studies on dogs confirmed the presence of endogenous antioxidants in the seminal plasma of pre-spermatic, spermatic, and post-spermatic fractions (11). Even so, a consistent body of literature reports that one of the main conditions that adversely affects sperm quality is an elevation of ROS, which in turn causes oxidative damage to sperm, following lipid peroxidation, DNA fragmentation and lesser concentrations of antioxidants (3, 14).

Recent evidence suggests that seminal microbiota may influence key characteristics of canine semen, prompting growing interest in the existence of a functional gut–testis axis (4, 15–17). Rather than acting as isolated ecosystems, microbial communities across body districts appear to be interconnected, with the gut microbiota potentially contributing to the microbial composition of distal sites, including the male urogenital tract (18). In this context, several studies across different animal species (e.g., bulls, poultry, turkeys, and dogs) have reported associations between specific bacterial taxa and semen quality parameters, suggesting a modulatory role of microbiota on reproductive function (15, 19–22). However, despite these advances, the current body of literature remains largely associative and fragmented. In particular, there is a lack of integrative studies clarifying whether and how gut microbial communities may influence testicular function and semen quality in dogs through defined biological mechanisms. Moreover, the extent to which gut-derived bacteria contribute to the composition and functional impact of seminal microbiota in this species remains unclear.

Thereby, in terms of nutrition and diet, providing gut health holds a pivotal role for the maintenance of microbiota balance, tissues homeostasis and redox status, also in testis environment. In particular, gut microbiota concurs to maintain a state of eubiosis, undertaking the key role of regulate oxidative metabolism, prevent pathogens’ colonization and modulate immune response (23–25). Moreover, regarding reproductive sphere, gut dysbiosis has been related to impaired spermatogenesis and sperm kinetics (26). Similarly, gut microbiota by influencing the host’s antioxidant defense system may modulate the activity of local oxidative stress-related enzyme, testosterone levels, and blood-testis barrier permeability (4, 24). Thus, an appropriate dietary plan is paramount to support breeders’ fertility by both ensuring a regular dietary intake of antioxidants and by balancing gut microbes composition (17, 27, 28). Therefore, the regulation of gut–testis axis by dietary manipulation has been suggested as a potential approach to managing fertility issues in mammals.

In this context, a growing body of evidence suggests that dietary probiotic supplementation may exert modulatory effects on male reproductive function, with particular relevance to sperm quality (27, 29). Probiotics are defined as live microorganisms that, when administered in adequate amounts, confer health benefits to the host, primarily through modulation of the gut microbiota (14, 30). Nevertheless, the mechanisms underlying their potential reproductive benefits remain incompletely elucidated. A hypothesis-driven framework suggests that these effects may be mediated through three interconnected pathways. First, probiotics may enhance systemic antioxidant defenses by stimulating the production of key enzymes such as glutathione and superoxide dismutase, thereby mitigating oxidative stress, a well-established contributor to impaired sperm function (4, 27). Moreover, probiotics may influence endocrine regulation, potentially modulating the hypothalamic–pituitary–gonadal axis and altering circulating levels of reproductive hormones. Also, probiotics may induce compositional and functional shifts in the gut microbiota, which in turn can affect systemic inflammation, metabolic homeostasis, and the gut–testis axis. Taken together, these mechanisms likely act in a coordinated manner, although their relative contribution may vary depending on the specific bacterial strains, host physiological status, and environmental stressors. Further targeted studies are required to disentangle these pathways and establish causality.

Moreover, previous findings have demonstrated the protective role of probiotic on sperm quality and reproductive physiology (4, 31, 32). It is reasonable hypothesized that probiotics’ efficiency in boosting semen quality may be addressed from a dual perspective: firstly, through the capacity of modulate of gut-testis axis (15), secondly through their potential antioxidant influence on sperm parameters, testicular histology, and testosterone level (14, 33, 34). Further, considering the implication of gut microbiota in endocrine signaling (17, 35), dietary probiotics might facilitate the establishment of a favorable environment for optimal spermatogenesis improving sperm quality parameters (27, 36, 39).

In order to explore the potential of dietary probiotics as valuable feed additives on dog’s health and fertility, this study evaluated the effects of a multi-strain probiotic mixture (Slab51®, SivoMixx®) on serum biochemical parameters, antioxidant status and seminal parameters in healthy adult male dogs.

SivoMixx® is a probiotic blend composed of strains of streptococcus, lactic acid bacteria and bifidobacteria already tested in companion and livestock species (37–39). In poultry and rabbit, the product enhanced growth performance, modulated caecal microbiota and ameliorated gut morphology (37, 38). While, in healthy dogs, multi-strain probiotic supplementation positively influenced intestinal microbiota and strengthened immune markers (39). Otherwise, commensal lactobacilli administration recorded positive results on dog sperm quality, including significant enhancement of kinematic parameters, acrosome integrity, and positively regulate expression of genes related with fertility and DNA repair (27, 36).

Currently, different researches have highlighted the potential of dietary manipulation as a way to maintain sperm cell fertility, and that oral antioxidant integration may improve oxidative status and quality of semen. Therefore, the present study focuses to clarifying the capacity of a multi-strain probiotic blend administration in optimizing semen parameters and oxidative status of healthy male dogs.

## 2 Materials and methods

### 2.1 Animals and study design

The trial was approved by the Ethics Committee of the DiMeV of University of Bari Aldo Moro, Italy (Approval no. 09/23, Prot. no. 1226-III/13). Procedures with animals were performed following the good veterinary practice for animal welfare according to national laws in force (D.Lgs 116/92) and informed owner consent was obtained.

The study was conducted on 14 clinically healthy owned male breeding dogs of different pure breeds, aged 2–4 years old and 10–30 kg of body weight with optimal body condition score (BCS) (!Supplementary Table S1 reported all recruited dogs characteristics). All subjects were previously submitted to clinical and ultrasonographic evaluation to assess health status and to exclude reproductive pathologies and were selected only those having physiological semen values.

**Figure 1:**
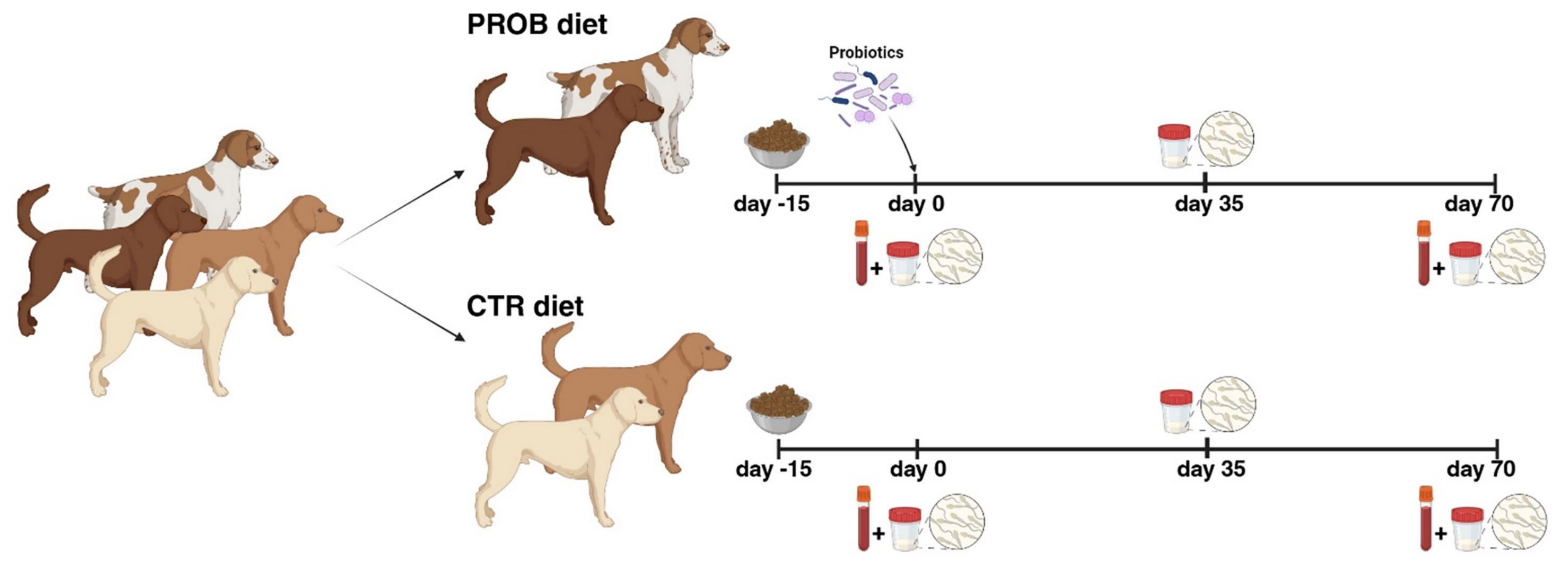
Experimental design involving dogs fed control (CTR) or probiotic (PROB) diet (created with BioRender.com).

Dogs were randomly divided into two groups: the control (CTR) group was fed without any additive, while the test group received a diet supplemented with a multi-strain probiotic (PROB) mixture. The Slab51® (marketed in Europe under the trademark SivoMixx®, Ormendes SA, Jouxtens-Mézery, CH) is a commercial multi-strain probiotic mixture containing 200 billion lactic acid bacteria per 1.5 g of product, comprised of the following strains: *Streptococcus thermophilus* DSM 32245/CNCM I-5570, *Bifidobacterium lactis* DSM 32246/CNCM I-5571, *Bifidobacterium lactis* DSM 32247/CNCMI-5572, *Lactobacillus acidophilus* DSM 32241/CNCM I-5567, *Lactobacillus helveticus* DSM32242/CNCMI-5573, *Lactobacillus paracasei* DSM 32243/CNCM I-5568, *Lactobacillus plantarum* DSM 32244/CNCM I-5569, and *Lactobacillus brevis* DSM 27961/CNCM I-5566. The probiotic was daily administered for 70 days *per os* immediately before the morning meal at a dose of 400 billion lyophilized bacteria as suggested by Rossi et al. (39). All subjects were fed a commercial dry adult maintenance diet, covering all nutrients’ requirements, twice daily and water was supplied *ad libitum*. Serum biochemical parameters and blood antioxidant status were collected at 0 and 70 days of experimental dietary supplementation. Before starting the feeding trial, semen collection was made at 15 days distance, as control, and then after 35 and 70 days from the beginning of the PROB dietary supplementation. The time of PROB administration was evaluated to cover an entire cycle of spermatogenesis, which in the dog lasts on average 62 days (40). All samples were collected during spring season to avoid possible effects due to the high temperatures of the summer season. The experimental design and sampling are reported in Figure 1.

### 2.2 Blood biochemistry and antioxidant status analyses

Blood samples were obtained aseptically by cephalic vein puncture using 22-gage needles and a negative-pressure 4 mL collection system for serum (without anticoagulant) and plasma (with 15 USP U/mL heparin) (Vacutainer®, Becton, Dickinson Canada Inc., Oakville, Canada). Tubes designated for plasma collection were centrifuged immediately (4,000 rpm for 20 min), whereas serum tubes were allowed to clot at refrigerated temperature for 10 min prior to centrifugation (4,000 rpm × 20 min). All plasma and serum aliquots were stored at −80 °C until analysis. Blood sampling was performed in the morning after a fasting period of at least 8 h.

Serum samples were used for the determination of clinical biochemistry parameters using an automated biochemistry analyzer (CS-300B; Dirui, Changchun, China). The analyzed parameters included total protein, albumin, blood urea nitrogen (BUN), creatinine, creatine phosphokinase (CPK), bilirubin, triglycerides, alanine aminotransferase (ALT), aspartate aminotransferase (AST), alkaline phosphatase (ALP), cholesterol, calcium, iron, potassium, magnesium, and sodium. Biochemical parameters and serum electrolytes were additionally determined using an automated analyzer (Alinity ci-series, Abbott, IL, USA).

Plasma samples were used for the assessment of oxidative stress markers and antioxidant enzyme activities. Plasma malondialdehyde (MDA) concentrations were determined using the thiobarbituric acid reactive substances (TBARS) assay, based on the reaction between MDA and thiobarbituric acid, yielding a pink chromogen with maximal absorbance at 535 nm (41). The activities of superoxide dismutase (SOD), catalase (CAT), and glutathione peroxidase (GSPx) were measured according to the methods described by Forte et al. (42). Serum total antioxidant capacity (TAC) was determined using a commercially available assay kit (Cayman Chemical, Ann Arbor, MI, USA), following the protocol reported by Michałek et al. (43).

### 2.3 Semen collection and sperm motility evaluation

Semen collection was performed by digital manipulation in the presence of a teaser bitch using an open collection system (Canine Collection Semen, Minitübe, Germany). The ejaculate was fractionated, and the three fractions were collected separately; the first fraction was discarded. The sperm-rich second fraction was used for the evaluation of sperm concentration and kinematic parameters, whereas the third fraction, corresponding to prostatic seminal plasma, was analysed for oxidative status. All analyses were conducted immediately after semen collection. Samples were maintained at room temperature, as this condition has been reported to better preserve sperm motility (44).

Sperm concentration and kinematic parameters of the second ejaculatory fraction were assessed using a computer-assisted sperm analysis (CASA) system (IVOS 12.0, Hamilton Thorne, USA). The evaluated parameters included total motility (%), progressive motility (%), distribution of motility classes (rapid, medium, slow, and static), sperm concentration (×10^6^/mL), straight-line velocity (VSL, μm/s), curvilinear velocity (VCL, μm/s), average path velocity (VAP, μm/s), amplitude of lateral head displacement (ALH, μm), beat-cross frequency (BCF, Hz), straightness (STR, %), and linearity (LIN, %). Immediately after collection, ejaculates were diluted in phosphate-buffered saline (PBS) to a final concentration of approximately 25 × 10^6^ spermatozoa/mL.

### 2.4 Seminal plasma antioxidant status determination

Reactive oxygen species (ROS) and biological antioxidant potential (BAP) concentrations in seminal plasma were determined using a photometric analytical system (FREE *Carpe Diem*®, Diacron International srl, Grosseto, Italy). The concentration of reactive oxygen metabolites (d-ROMs) was measured using a free radical elective evaluator (FREE *Carpe Diem*®, Diacron International), equipped with a spectrophotometric reader and specific assay kits (d-ROMs test; Wismerll Co. Ltd., Tokyo, Japan), optimized for use with the FREE *Carpe Diem*® system according to the manufacturer’s instructions. Briefly, 20 μL of seminal plasma was mixed with 1 mL of buffered solution (R2 reagent, pH 4.8) in a cuvette, followed by the addition of 20 μL of chromogenic substrate (R1 reagent). After thorough mixing, the cuvette was incubated in the thermostatically controlled block of the analyzer at 37 °C for 5 min, and absorbance was measured at 505 nm. Results were expressed in arbitrary units (U/CARR), with one unit corresponding to 0.8 mg/L of hydrogen peroxide.

The BAP was assessed using the same free radical elective evaluator (FREE *Carpe Diem*®) and dedicated assay kits (BAP test; Wismerll Co. Ltd.), optimized for the system and applied according to the manufacturer’s protocol. Briefly, 50 μL of chromogenic substrate (R2 reagent) was mixed with 1 mL of reactive solution (R1 reagent) in a cuvette, and baseline absorbance at 505 nm was recorded. Subsequently, 10 μL of seminal plasma was added, mixed thoroughly, and incubated at 37 °C for 5 min in the analyzer block, after which absorbance at 505 nm was recorded. Results were expressed as mmol/L of reduced ferric ions.

Total antioxidant capacity (TAC) was determined according to the method described by Domosławska et al. (45), based on the ferric-reducing antioxidant power of the samples. The working reagent, consisting of 300 mmol/dm^3^ acetate buffer (pH 3.6), 10 mmol/dm^3^ 2,4,6-tripyridyl-s-triazine in 40 mmol/dm^3^ HCl, and 20 mmol/dm^3^ FeCl₃·6H₂O mixed in a 10:1:1 ratio, was prepared immediately before use. An aliquot of 2,250 μL of working reagent was mixed with 25 μL of sample, and absorbance at 593 nm was measured against the working reagent blank. After exactly 10 min of incubation at room temperature, absorbance was measured again. The difference in absorbance between 0 and 10 min was compared with a standard curve generated using Fe(II) solutions ranging from 0 to 1,000 μmol/dm^3^. Results were expressed as μmol per g of protein in the sample.

### 2.5 Statistical analysis

Data were tested for normality and homogeneity of variance prior to statistical analysis, in accordance with standard guidelines for biological datasets, to ensure the suitability of the applied models. Baseline differences between the control and experimental groups were initially assessed using one-way ANOVA to verify the effectiveness of randomization and confirm group comparability across all evaluated parameters. Longitudinal semen and biochemical data were analyzed using linear mixed-effects models to account for repeated measurements within subjects. In these models, treatment, time, and their interaction were included as fixed effects, while individual animal was treated as a random effect to account for within-subject correlation. When relevant, additional fixed factors (i.e., breed) were included to control for potential sources of biological variability. Model assumptions were verified by inspection of residual distributions. When a significant main effect or interaction was detected, *post hoc* comparisons at individual time points were performed using one-way ANOVA with appropriate adjustment for multiple testing. All statistical analyses were conducted using Cohort Stat software (2006 version 6.311; Cohort Software Inc., Monterey, CA), and statistical significance was set at *p* < 0.05.

## 3 Results and discussion

In healthy dogs, suboptimal semen quality may result in low fertility potential, thus monitoring canine seminal parameters has become the standard procedure for rating fertility efficiency in dog breeders.

Recent data shown that subfertility in dogs is responsible for male fertility impairment in 30% of cases (36, 46), consequently optimizing seminal parameters is one of the primary intents of breeding programs. To date, the etiology of suboptimal semen quality has a multifactorial origin (47) and may result from external factors, such as environment (48), diet (49, 50) and stress (51), as well as internal factors such as aging (52), metabolism (53), nutrient availability (54, 55), oxidative status (47, 48), endocrine and hormonal disorders, urogenital tract affections (56, 57).

Although male infertility is multifactorial, there are supporting evidences that it is closely correlated with seminal oxidative stress, resulting in changes in sperm qualitative and quantitative parameters such as sperm morphology, kinematics and DNA integrity (3, 34, 58). Further, since redox status could also affect gut microbiota balance and given its link with the testicular function, it is reasonable hypothesized that manage gut microbiota through dietary manipulation, may boost sperm quality (17, 36). Therefore, this study aimed to investigate the influence of a probiotic multi-strain mixture (Slab51®, Sivomixx®) on plasma and seminal parameters, also deepening the relation between oxidative status and quality of semen in healthy adult breeder dogs.

Hematology and serum biochemistry are dynamic systems strictly linked to animals’ nutrition and their internal and external environment (59, 60). In this study, the effects of dietary probiotic on blood biochemistry of breeder dogs were evaluated by comparing results at 0 and 70 days from the begin of the experimental feeding period (Tables 1, 2). Compared to the initial serum biochemical and mineral profile, at 70 days, BUN, triglycerides and total cholesterol levels resulted significantly reduced by PROB supplementation, while the remaining blood parameters did not differ between groups. The improvement in lipid profile has been reported in many investigations on dietary probiotics. In a recent study, employing the multi-strain probiotic Sivomixx® registered a significant decrease in total cholesterol, triglycerides, LDL and HDL, main indicators of lipid metabolism and lipid transport in the body (38). Similarly, a synbiotics preparation with *Lactobacillus acidophilus* and inulin in dog diet maintained physiological levels of red blood cells, white blood cells and platelets, while boosted hemoglobin level and significantly reduced HDL-cholesterol (61). Lower value of triglyceride and total cholesterol was found by Kumar et al. (59) in dogs fed a diet supplemented with specie-specific *Lacotbacillus jhonsonii* (CPN232), a canine-origin probiotic. Literature reported that probiotics’ hypocholesterolemic effect resulted from a multilevel action. These complexes may incorporate cholesterol into their cells and use it for cellular metabolism, making short fatty acids (SCFAs) (62). In turn, SCFAs of microbial fermentative derivation may inhibit liver cholesterol synthesis or plasma cholesterol mobilization to liver (63). Moreover, gut bacteria prevent cholesterol formation and intestinal absorption by promoting bile salts deconjugation (59).

**Table 1:** Effect of dietary supplementation of multi-strain probiotic on blood biochemistry of male dogs.

Item	Group	Time (days)		SEM	*p*-value		
		0	70		Group	Time	Group × time
Total protein (g/dL)	CTR	6.83	6.40	0.404	ns	ns	ns
	PROB	6.92	6.56				
Albumin (g/dL)	CTR	3.35	3.30	0.390	ns	ns	ns
	PROB	3.22	3.14				
BUN (mg/dL)	CTR	39.50	38.00	0.887	**	*	*
	PROB	38.20	33.40				
Creatinine (mg/dL)	CTR	1.24	1.27	0.091	ns	ns	ns
	PROB	0.98	1.06				
CPK (IU/L)	CTR	69.00	59.00	1.221	ns	ns	ns
	PROB	70.40	58.20				
Bilirubin (mg/dL)	CTR	0.13	0.10	0.011	ns	ns	ns
	PROB	0.10	0.12				
Triglycerides (mg/dL)	CTR	69.17	71.00	1.942	*	*	*
	PROB	73.60	66.80				
ALT (IU/L)	CTR	37.67	39.00	1.002	ns	ns	ns
	PROB	38.60	35.00				
AST (IU/L)	CTR	24.83	23.00	0.663	ns	ns	ns
	PROB	25.60	26.40				
ALP (IU/L)	CTR	37.00	35.00	0.901	ns	ns	ns
	PROB	45.20	44.40				
Cholesterol (mg/dL)	CTR	216.8	223.0	14.05	**	*	*
	PROB	228.8	209.4				

*p < 0.05; **p < 0.01; ns, not significant. CTR, control diet; PROB, diet including multi-strain probiotic; BUN, blood urea nitrogen; CPK, creatinine phosphokinase; ALT, alanine aminotransferase; ASP, aspartate aminotransferase; ALP, alkaline aminotransferase.

**Table 2:** Effect of dietary supplementation of multi-strain probiotic on blood biochemistry of male dogs.

Item	Group	Time (days)		SEM	*p*-value		
		0	70		Group	Time	Group × time
Calcium (mg/dL)	CTR	9.82	10.30	0.442	ns	ns	ns
	PROB	9.96	9.98				
Iron (mg/dL)	CTR	191.6	183.0	19.78	ns	ns	ns
	PROB	205.8	192.2				
Potassium (mg/dL)	CTR	4.47	4.80	0.374	ns	ns	ns
	PROB	4.50	4.56				
Magnesium (mg/dL)	CTR	2.19	2.00	0.290	ns	ns	ns
	PROB	2.01	1.99				
Sodium (mmol/L)	CTR	148.8	146.0	16.02	ns	ns	ns
	PROB	144.6	142.8				

ns, not significant; CTR, control diet; PROB, diet including multi-strain probiotic.

Several studies have shown that probiotic microorganisms exhibited an antioxidant promoting activity, also endorsing the production of specific non-enzymatic antioxidant biomolecules and enzymes like the chief free-radical scavenger glutathione (61, 64). Regarding the serum oxidative status of dogs tested in this trial, after 70 days, compared to control, probiotic group showed a remarkable increase (*p* < 0.001) of the endogenous antioxidant enzymes (GSPx, CAT and SOD) and total antioxidant capacity (TAC), whereas MDA value did not register differences between tested groups (Table 3). Previous evidences have detected that *Lactobacillus* and *Bifidobacterium* could exert antioxidant activities and reduce oxidative damage both *in vivo* and *in vitro* (65). Findings suggested that the use of probiotics prevents oxidative stress by significantly improving TAC and MDA levels (61, 66–68).

**Table 3:** Effect of dietary supplementation of multi-strain probiotic on blood antioxidant status of male dogs.

Item	Group	Time (days)		SEM	*p*-value		
		0	70		Group	Time	Group × time
MDA (μmol/L)	CTR	3.04	3.79	0.390	ns	ns	ns
	PROB	3.09	3.61				
SOD (U/mL)	CTR	38.81	36.95	1.052	***	***	***
	PROB	38.56	46.55				
CAT (U/mL)	CTR	1.82	1.88	0.131	***	**	***
	PROB	1.85	2.64				
GSPx (nmol NADPH ox/mL)	CTR	2.21	1.80	0.073	***	***	***
	PROB	2.13	3.25				
TAC (mmol/L)	CTR	0.28	0.21	0.034	***	***	***
	PROB	0.25	0.41				

**p < 0.01; ***p < 0.001; ns, not significant; CTR, control diet; PROB, diet including multi-strain probiotic; MDA, malonaldehyde; SOD, superoxide dismutase; CAT, catalase; GSPx, glutathione peroxidase; TAC, total antioxidant capacity.

The results of this study align with the outlined impact of probiotics on controlling and mitigating oxidative stress processes. It is noteworthy that oxidative stress, characterized by an imbalance between ROS production and antioxidants systems, is closely linked to the health of organisms (69) and that probiotics are found to have a part in protect gut environment from oxidative products (59, 64, 70, 71). Notably, in healthy dog, dietary supplementation with Slab51® probiotic mixture modulated the composition of the intestinal microbiota also enhancing systemic and mucosal immune response (39). Recently, Tufarelli et al. (38) found that feeding rabbit with Sivomixx® exerted a remarkable effect on both antioxidative enzymes activity, caecal microbiota population and gut heath parameters. These outcomes can be traced back to the ability of probiotics in reshaping the intestinal microbiota, and to the influence of this “hidden organ” on both local and systemic oxidative status. A large body of evidences suggested that probiotics’ mechanism of action can be seen from two different but overlapping angles: first of all, by colonizing gut environment, probiotics modulate microbial functions and in turn influence the gut-organs axis (4, 72). In particular, gut microbiota and its metabolites can regulate multiple systems other than gut, including the urogenital tract (18). Secondly, they play a central antioxidant action that is able to strengthen body oxidative balance. For instance, following probiotic supplementation gut microbiota modulation has been proven to hinder oxidative processes and positively influence testicular function by preserving seminiferous tubule structure and increasing spermatogonial stem cells (4, 73). Accordingly, probiotics by regulating systemic ROS level may consequently reduce oxidative processes at local level. Previous studies in canines suggested a correlation between systemic oxidative status and endogenous antioxidants concentration in semen (11, 74). Otherwise, the influence of systemic and seminal oxidative status on semen quality parameters has been already reported in humans (75). In particular, it was demonstrated that despite a certain amount of ROS is essential for spermiogenesis, excessive ROS can induce oxidative stress, thus leading to sperm damage. Therefore, as the interplay among diet, gut microbiota and semen oxidative balance has been established (27, 76–78), the present study investigated the influence of probiotics intake not only on blood antioxidant status but also on dog seminal plasma.

Data gathered in this study on dog seminal oxidative status are reported in Table 4. After 35 and 70 days of treatment, comparing seminal concentrations between the two groups, it was found a lower ROS production in dogs fed PROB (*p* < 0.001) and a significant improvement of BAP and TAC values compared to the control group. Regarding ROS concentration, there were found significant effects due to group (*p* < 0.001), timepoints (*p* < 0.01) and their interaction (*p* < 0.01). Similarly, the BAP test showed significant differences due to groups, timepoints, and the interaction group × time (*p* < 0.001, *p* < 0.01, and *p* < 0.05 respectively). Lastly, a strong significant improvement (*p* < 0.001) of the seminal TAC was detected in dogs under PROB treatment.

**Table 4:** Effect of dietary supplementation of multi-strain probiotic on seminal plasma antioxidant status of dogs.

Item	Group	Time (days)			SEM	*p*-value		
		0	35	70		Group	Time	Group × time
ROS (U/Carr)	CTR	148.7	173.5	215.0	17.22	***	**	**
	PROB	149.5	154.3	157.1				
BAP (μmol)	CTR	1,410	1,247	1,105	79.4	***	**	*
	PROB	1,387	1,310	1,277				
TAC (μmol/g protein)	CTR	12.61	12.89	11.02	1.461	***	***	***
	PROB	13.02	14.38	17.13				

*p < 0.05; **p < 0.01; ***p < 0.001; ns, not significant; CTR, control diet; PROB, diet including multi-strain probiotic; ROS, reactive oxygen species; BAP, biological antioxidant potential; TAC, total antioxidant capacity.

Semen redox balance regulates sperm cells integrity, maturation, and kinematics, overall seminal quality parameters. Thus, in this feeding trial dog seminal quality was tested to evaluate the influence of probiotic integration on fertility efficiency.

In Tables 5, 6 are reported the mean values of semen quality and motility parameters. Significant differences were found for sperm concentration (*p* < 0.001), VAP (*p* < 0.05), VSL (*p* < 0.05), VCL (*p* < 0.01), ALH (*p* < 0.05), and LIN (*p* < 0.001) between the experimental PROB dietary treatment and the control. Conversely, the supplementation with multi-strain probiotic had no effect on BCF and STR. A gradual enhancement of total motility and progressive sperm in PROB dog group was observed at all time points, with significant effect due to group (*p* < 0.001), timepoint (*p* < 0.01) and their interaction (*p* < 0.01), respectively. The same significant effects were detected for the other motility parameters, with the exception of static % resulting unaltered between groups (*p* > 0.05).

**Table 5:** Effect of dietary supplementation of multi-strain probiotic on semen quality characteristics of dogs.

Item	Group	Time (days)			SEM	*p*-value		
		0	35	70		Group	Time	Group × time
Concentration (×10^6^/mL)	CTR	160.4	183.3	118.9	23.23	***	***	***
	PROB	147.8	215.2	318.6				
VAP (μm/s)	CTR	147.6	163.0	173.1	12.54	*	*	*
	PROB	139.7	148.8	164.9				
VSL (μm/s)	CTR	134.1	139.3	160.1	10.05	*	**	ns
	PROB	127.2	127.0	145.7				
VCL (μm/s)	CTR	172.5	247.5	225.5	20.67	**	*	*
	PROB	182.0	178.9	201.6				
ALH (μ)	CTR	6.48	8.80	6.90	0.961	*	*	ns
	PROB	5.97	5.46	5.96				
BCF (Hz)	CTR	28.38	26.90	29.70	5.460	ns	ns	ns
	PROB	23.87	22.90	26.88				
STR (%)	CTR	89.22	84.00	90.00	1.332	ns	ns	ns
	PROB	89.50	88.00	89.00				
LIN (%)	CTR	64.38	58.00	68.00	6.878	***	**	*
	PROB	70.25	68.25	72.50				

*p < 0.05; **p < 0.01; ***p < 0.001; ns, not significant; CTR, control diet; PROB, diet including multi-strain probiotic; VAP, average path velocity; VSL, straight-line velocity; VCL, curvilinear velocity; ALH, amplitude of lateral head displacement; BCF, beat-cross frequency; STR, straightness; LIN, linearity.

**Table 6:** Effect of dietary supplementation of multi-strain probiotic on semen quality characteristics of dogs.

Item	Group	Time (days)			SEM	*p*-value		
		0	35	70		Group	Time	Group × time
Total motility (%)	CTR	91.50	93.50	97.00	9.111	**	***	**
	PROB	92.00	94.50	98.75				
Progressive motility (%)	CTR	60.70	65.00	69.00	8.002	***	**	**
	PROB	58.82	69.25	71.75				
Rapid (%)	CTR	77.30	78.00	75.00	8.676	**	**	**
	PROB	66.25	76.50	79.50				
Medium (%)	CTR	5.67	4.00	4.00	1.855	**	**	*
	PROB	9.50	12.00	8.75				
Slow (%)	CTR	9.67	8.00	18.00	1.977	**	**	**
	PROB	16.00	6.75	10.25				
Static (%)	CTR	7.33	1.00	2.00	2.403	ns	ns	ns
	PROB	8.00	1.00	1.50				

*p < 0.05; **p < 0.01; ***p < 0.001; ns: not significant. CTR: control diet; PROB: diet including multi-strain probiotic.

Maintaining basic cellular functions requires a fine balance between ROS production and antioxidant systems (11, 79). In reproductive processes, basal levels of ROS are intrinsic and play an essential role entailing the modulation of sperm maturation and capacitation, acrosome activation, and sperm–oocyte fusion (10). Conversely, sperm cells are very sensitive to oxidative processes due to the high content of unsaturated fatty acids in their membranes (3, 80). Due to this, in the seminal environment ROS levels are controlled by a specific set of endogenous and exogenous antioxidants (81), that contribute to avoid ROS overload and preserve semen quality (11). In particular, the levels of catalase, glutathione peroxidase, and exogenous antioxidants play an important role in sperm maturation, quality parameters, and fertility (27, 48, 82). Hypofertile dogs with poor semen quality showed higher serum ROS levels and lower antioxidant enzymes value than fertile dogs (3, 11). Hence, the oxidative cascade reduces sperm quality and causes serious consequences, such as peroxidative damage and loss of membrane integrity, decrease in adenosine triphosphate (ATP) concentrations or inhibition of enzyme activity, as well as DNA fragmentation responsible for mutations, metabolic failure and apoptosis. Additionally, it has been reported that enhanced free radicals may influence sperm count, viability and kinetic parameters (79, 83). Indeed, oxidative alterations by depleting ATP levels and modifying tail’s membrane impair sperm motility and its ability to fuse with the oocyte (34, 84). Therefore, the antioxidant component of seminal plasma is essential to maintain the balance of free radicals’ production in order to prevent spermatic oxidative damage.

Results of the present study demonstrated a favorable influence of Sivomixx® blend administration on both seminal oxidative status and quality parameters, confirmed by the lower concentration of ROS in the semen of PROB treated breeder dogs and a significant improvement of the antioxidative capacity (BAP and TAC) of the same group when compared to the control. The enhancement of semen antioxidant status is reflected in quality parameters of semen that was found significantly improved in PROB group; thus, suggesting that dietary probiotics exerted a protective effect on motility and viability of sperm cells, boosting their fertility features.

Accumulating evidences from human and animal studies provided novel insights into the role of dietary antioxidants in safeguarding reproductive function and the connection between oxidative status and semen quality parameters. In dog, it has been shown that probiotic administration increased glutatione peroxidase (GPX) levels and sperm kinematic parameters, overall improving semen quality (27). Probiotic efficiency on boosting male fertility may be related to the restoration of the adverse effect of oxidative stress induced by fatty diet and aging (14, 52). In studies conducted on rats fed high-fat diets, the supplementation with probiotics restored SOD and GSH-Px activities and reduced MDA levels, while reduced the level of oxidative damage, thus improving sperm quality (79, 85). Oral administration of commensal Lactobacillus spp. on healthy normozoospermic dogs was shown to improve total and progressive motility, acrosome integrity, and other kinematic parameters in seminal sample of treated group (36). The authors supposed that the enhancement of these parameters might be due to the antioxidant effects of lactobacillus, as previously found in humans by Barbonetti et al. (33). Furthermore, a mixture of *L. acidophilus, L. bulgaricus, L. rhamnosus, L. casei, Bifidobacterium breve, B. longum, and Streptococcus thermophilus* significantly enhanced sperm concentration, motility, DNA integrity, and seminal lipid peroxidation after 80 days of supplementation in men (86).

Findings of this study confirmed that probiotics supplementation is effective in fostering blood and seminal oxidative capacity, which in turn optimizes seminal plasma parameters in normozoospermic dogs, also positively influencing fertility capacity. Further, in the present trial, since probiotics administration covered the entire spermatogenic cycle duration of about 62 days (40), data analysis at different timepoints, consented to deduce that at 70 days, the probiotics supplementation was effective to influence significantly oxidative status and sperm quality already after one spermatogenesis cycle in dogs.

## 4 Conclusion

Male canine fertility can be influenced by both endogenous and exogenous factors, leading to fluctuations in semen quality that may ultimately affect reproductive fitness. Moreover, the widespread use of artificial insemination in breeding programs has increased the demand for consistently high-quality semen. In both chilled and frozen–thawed spermatozoa, superoxide leakage represents a common challenge, impairing sperm function and fertilizing capacity. Our findings indicate that probiotic supplementation enhances seminal antioxidant defences, mitigates excessive reactive oxygen species production, and protects spermatozoa from oxidative damage, thereby representing a promising strategy to optimize semen characteristics and support fertility. The present study evaluated the effects of a multi-strain probiotic blend on systemic and seminal oxidative status, as well as on semen quality parameters, in healthy breeding dogs over time. Significant improvements were observed across all evaluated parameters in the group receiving the probiotic-supplemented diet, which included *Lactobacillus*, *Bifidobacterium*, and *Streptococcus* strains (Sivomixx®). The results of our feeding trial provide compelling evidence supporting the efficacy of dietary multi-strain probiotic supplementation in enhancing oxidative balance and sperm quality in breeding dogs. These findings reinforce the hypothesis that probiotic administration exerts antioxidant effects, protecting sperm membrane integrity and DNA from ROS-induced damage, with consequent improvements in seminal parameters. Furthermore, evidence suggests that probiotics may likely alter semen parameters through their modulation of gut microbiota. Accordingly, further investigations are underway to characterize potential changes in the canine gut microbiota following dietary supplementation with the Sivomixx® probiotic blend. Such additional evaluations may help clarify the putative gut–testis axis and its role in fertility, paving the way for a natural and targeted strategy to support male reproductive health in dogs. However, despite these encouraging findings, some limitations should be acknowledged, including the relatively small cohort size, the absence of integrated microbiome and endocrine analyses, and the lack of longitudinal fertility outcomes, which collectively limit the ability to establish causality and broader applicability, which warrant further investigation in larger and more comprehensive studies.

## Data Availability

The raw data supporting the conclusions of this article will be made available by the authors, without undue reservation.
