# Supplementary material for: Nutritional modulation of the gut–reproductive axis: multi-strain probiotic blend on oxidative and seminal parameters in healthy male dogs
Source: Front Vet Sci. 2026 Apr 24;13:1820546. doi: 10.3389/fvets.2026.1820546 (PMC13152741; doi:10.3389/fvets.2026.1820546)
Supplement: Supplementary file 1 [file Table_1.pdf]

Table S1. Breeds of dogs in control (CTR, n = 7) and experimental probiotic (PROB, n = 7) group.

Item	Breed	Age	Body weight, kg	BCS
Control (CTR)	Border Collie	2	21	3.0
	French Bulldog	2	13	3.5
	Golden Retriever	3	30	3.0
	Cocker Spaniel	4	15	3.0
	West Highland White Terrier	3	10	3.5
	Mixed breed	4	29	3.5
	Poodle (Standard)	3	20	3.0
Probiotic (PROB)	Dalmatian	4	26	3.0
	Mixed breed	2	10	3.0
	Weimeraner	4	30	3.0
	Cocker Spaniel	3	13	3.0
	French Bulldog	3	11	3.0
	Samoyed	2	25	3.5
	Setter (English)	3	21	3.0
